# Using Deep RNA Sequencing for the Structural Annotation of the Laccaria Bicolor Mycorrhizal Transcriptome

**DOI:** 10.1371/journal.pone.0009780

**Published:** 2010-07-06

**Authors:** Peter E. Larsen, Geetika Trivedi, Avinash Sreedasyam, Vincent Lu, Gopi K. Podila, Frank R. Collart

**Affiliations:** 1 Biosciences Division, Argonne National Laboratory, Lemont, Illinois, United States of America; 2 Department of Biological Sciences, University of Alabama in Huntsville, Huntsville, Alabama, United States of America; Virginia Tech, United States of America

## Abstract

**Background:**

Accurate structural annotation is important for prediction of function and required for *in vitro* approaches to characterize or validate the gene expression products. Despite significant efforts in the field, determination of the gene structure from genomic data alone is a challenging and inaccurate process. The ease of acquisition of transcriptomic sequence provides a direct route to identify expressed sequences and determine the correct gene structure.

**Methodology:**

We developed methods to utilize RNA-seq data to correct errors in the structural annotation and extend the boundaries of current gene models using assembly approaches. The methods were validated with a transcriptomic data set derived from the fungus *Laccaria bicolor*, which develops a mycorrhizal symbiotic association with the roots of many tree species. Our analysis focused on the subset of 1501 gene models that are differentially expressed in the free living vs. mycorrhizal transcriptome and are expected to be important elements related to carbon metabolism, membrane permeability and transport, and intracellular signaling. Of the set of 1501 gene models, 1439 (96%) successfully generated modified gene models in which all error flags were successfully resolved and the sequences aligned to the genomic sequence. The remaining 4% (62 gene models) either had deviations from transcriptomic data that could not be spanned or generated sequence that did not align to genomic sequence. The outcome of this process is a set of high confidence gene models that can be reliably used for experimental characterization of protein function.

**Conclusions:**

69% of expressed mycorrhizal JGI “best” gene models deviated from the transcript sequence derived by this method. The transcriptomic sequence enabled correction of a majority of the structural inconsistencies and resulted in a set of validated models for 96% of the mycorrhizal genes. The method described here can be applied to improve gene structural annotation in other species, provided that there is a sequenced genome and a set of gene models.

## Introduction

Advances in sequencing technology have led to an improved appreciation of the biological diversity associated with specific ecosystems and the complexity of the molecular systems involved in the perception and response to external stimuli. Mapping these signaling and response pathways is especially challenging in sequence data sets from environmental sequencing projects where uncharacterized organisms often represent a high proportion of the sequence data. The standard mechanisms for functional and structural annotation of genomes are less than perfect and prediction methods that rely on sequence homology have limitations for inferring the structural and functional properties of some genes [1], [2], [3]. Even proteins with homologs of known function may have a different biological role due to altered regulation, posttranslational modification, or cellular compartmentalization [4], [5]. Estimates vary, but there is a general consensus that 50–70% of ORFs in newly sequenced genomes are described as having unknown or poorly characterized function [1], [6], [7]. Increased understanding of the biological information content in the sequence data will require a combination of bioinformatic and experimental approaches to characterize the function of these gene products.

Experimental approaches for functional characterization of gene products are often compromised by incorrect or insufficient knowledge of the gene structure to enable extraction of the protein sequence for experimental studies. To facilitate the process for acquisition of function from complex sequence data sets, we have developed methods to utilize RNA-seq data to detect and correct errors in the structural annotation. Software tools have been developed that perform spliced alignments of EST sequences to DNA [8], [9] or map RNA-Seq reads [10] to recover splice junctions. Our approach delineates the exon/intron structure necessary to extract the protein coding sequence for experimental and homology based approaches for functional interrogation. We also evaluated methods to extend the boundaries of current gene models using assembly approaches applied to the RNA-seq data. Both of these approaches support accurate structural annotation which is a requirement for experimental approaches for functional characterization and also provides validated sequence to utilize tools that enable identification cellular localization signals, domains. Structural annotation of genes in sequenced genomes from basidiomycetes is a challenge due to the high intron density and the frequent presence of small exons coding for less than 10 amino acids. The process developed for validation and improvement of the structural annotation can be applied on a genome scale to support experimental studies of the molecular events associated with mycorrhizal symbiosis as well as facilitate better comparative genomics and evolutionary analyses of gene families and their functions.

To validate our proposed system, we used a transcriptomic data set derived from the fungus *Laccaria bicolor*. This fungus is a member of the Basidiomycota and its intron density and distribution of small introns provide a challenge for structural annotation [11], [12], [13]. *L. bicolor* develops a mutualistic symbiotic association with many tree species [14], [15]. This complex association of the fungus and the plant roots provides nutritional benefits to both partners [15]. The fungi contribute phosphorous, nitrogen and mobilized nutrients from organic matter and in return the fungus obtains plant-derived carbohydrates [16], [17]. In mycorrhizal systems, up to 25% of the plant photosynthate is utilized by the fungal partner [18]. This fungal-plant symbiosis is a widespread process of major ecological importance. Knowledge of the molecular events associated with the development of the mycorrhizal system is essential for our understanding of natural biological processes related to carbon sequestration, carbon management, sustainability and bioenergy.

## Results

### Method Overview

**Figure 1:**
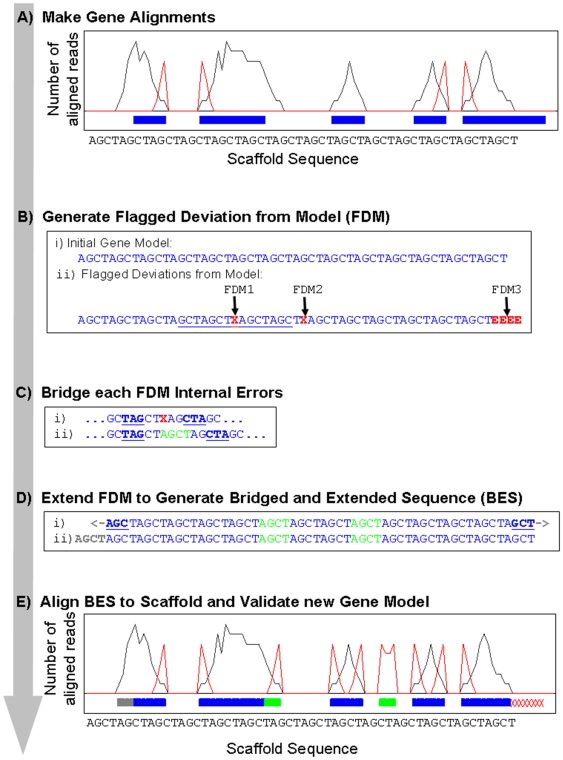
Overview of the structural annotation analysis method. In the illustrations, the original gene model is shown in blue, the number of aligned reads at a particular bp in the scaffold is in grey, and the number of reads that align to the gene model but not to the scaffold are in red. A) Bowtie is used to align RNA-seq reads and identify regions where expressed RNA sequence does not match predicted gene model. B) Errors in the gene model are identified where there is a predicted intron (i = predicted expressed gene sequence) but no spanning expressed sequence (“1” and “2” in example above) or where there is predicted gene but no sequence alignments (“3” in above example). The resulting flagged deviation from the model (FDM) is tagged with ‘X’ to indicate the presence of an internal error in the gene model or and an ‘E’ to indicate an error at the beginning or end of the gene model as illustrated by the expressed sequence string in Dii. C) For each internal error (red X) in FDM, such as error “1” in (A), an 18-mer ‘probe’ sequence is generated up and downstream of error (underlined and bold sequence in (Ci). This intron-spanning sequence was used to search the set of RNA-seq reads. for contiguous expressed sequence between selected probes (Green sequence in (Cii).) D) Error from beginning and end of FDM (such as Error “3” in above example) are removed by probes sequences generated from beginning and ends of expressed gene sequence (Bold and underlined sequence in example Di). These probes are used to search for expressed contiguous sequences to extend the gene model up and downstream (Grey sequence in Dii). The merged identified expressed sequence with correctly ascribed splice sites is the Bridged and Extended Sequence expressed (BES). E) The BES (Dii) is aligned to scaffold sequences to determine gene structure. In cartoon below, unchanged gene model is in blue, internal additions to gene model is in green, extensions up or downstream of the model are grey, and deletions from the initial gene model are indicated by red ‘X’s.

Our approach for validation and/or correction of gene models is organized in a framework of five conceptual components (Figure 1). The initial process uses Bowtie [19] to align RNA-seq reads to the set of gene models (BestModels v1.0 available for download at the Joint Genome Institute [JGI], denoted as BestModelsv1 in the text) and the genome (laccarria.allmasked available for download at the JGI). The outcome of this process for the set of transcript sequences which aligned to both the scaffold and the gene models is the generation of a graphical representation (Figure 1, A) and the identification of regions where expressed RNA sequence does not match predicted gene model. The algorithm described in the Methods Section enables analysis of the aligned reads and the identification of sequence incongruities between the predicted gene models and the observed transcriptomic data (Figure 1, B). These sequence discrepancies are saved as Flagged Deviations from the Model (FDM) sequences and provide sufficient information for sequence reconstruction using the transcriptomic reads. Sequence discrepancies internal to the gene model are corrected by identification of bridging sequences that span introns or contiguous reads that eliminate inserted introns (Figure 1, C). A slight modification of this approach addresses FDM at the beginning or end of the gene model and enables extension of the sequence upstream or downstream or the original gene model termini (Figure 1, D). The outcome of these successive processes is a contiguous Bridged and Extended Sequence (BES) which is validated by alignment to the genome scaffold sequences (Figure 1, E).and used to generate the gene model's corrected structural annotation. The gene structure with the correctly identified splice regions is derived from the scaffold alignment and saved in a General Feature Format (gff) file. Revised gene models and modified transcript sequences derived from this study are provided as supporting data in gff format (Text S1) and fasta format (Text S2), respectively. It should be noted that our method to utilize RNA-seq data to correct errors in the structural annotation and extend the boundaries of current gene models does not require specification of splice donor or acceptor sites. A consequence of this approach is increased ambiguity of the exact genome location of the splice site in the alignments of the transcript-derived models to the genome. This process can lead to the improper placement of the final coding nucleotide associated with the beginning or end of an exon. This can be resolved by manual inspection of the gene of interest or the output of this method can also be used in conjunction with other splice assembly programs. A list of all project data files and resources are available for viewing and download via the project URL provided in data resources file Text S3.

### Evaluation of optimal sequencing depth parameters for gene model validation

**Figure 2:**
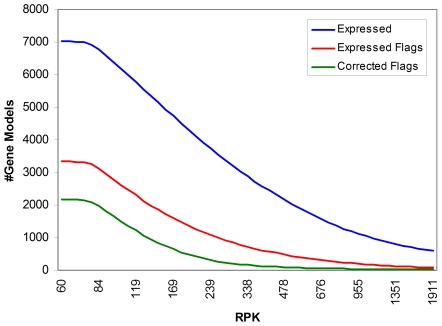
Relationship between the RPK expression levels and gene model characteristics. The traces represent the total number of gene models expressed (blue line) at or above some minimum RPK (log scale), the number of models in the subset of expressed genes that have at least one detectable internal sequence deviation (red line), and total number of expressed models that still contain at least one detectable internal sequence deviation after attempts were made to bridge those flags with the set of expressed RNAseq data (green line).

The algorithm described in the Methods Section enables analysis of the aligned reads and the identification of sequence incongruities between the predicted gene models and the observed transcriptomic data. These discrepancies are noted as flagged deviations from the BestModelsv1 (FDM) sequence and can either represent regions of the predicted model sequence with no transcriptomic sequence coverage or actual errors in the structural annotation. To select a useful minimum expression threshold for validation of this method and confidant correction of gene models, we evaluated the number of FDM sequences for all gene models expressed in our data set at a range of expression levels (Figure 2). 7023 gene models or 34% of all best gene models were expressed at a reads per Kb of gene sequence (RPK) of 60 or greater in the transcriptomic data set. A RPK of 60 represents an average sequence depth of 3; the human genome sequence standard for 99% accuracy (http://www.genome.gov/10000923). This set of 7023 expressed genes was used to identify FDM sequences and contiguous expressed sequence spanning the internal flags was generated. The analysis was restricted to the sequence coverage of the original gene models and flags at the termini of gene models were not considered in this analysis. This restriction was imposed to avoid erroneous interpretations that arise by failure to extend a model beyond its predicted termini. For example, identification of expressed sequence that bridges two probe sequences can be judged a success. However the failure to detect expressed extended sequence may result from an appropriate truncation of the predicted model or may simply arise as a consequence of insufficient transcript coverage.

At an RPK of 60, no sequence deviations were detected for 3188 or 53% of the expressed gene models. As expected, the number of gene models with no detectable sequence deviations shows an inverse relationship to the depth of transcript sequence coverage (Figure 2). The use of the transcript data to identify and correct FDM sequences result in a substantial increase in the total number of the models without flags (indicated by the green trace in Figure 2). At the same RPK, the difference between the numerical values of the green and red traces represents the number of corrected models. It should be noted that this represents a significant under-representation of the efficacy of our proposed method, as only models with zero flags after correction are considered a success. For example the gene model encoding protein ID 322344 expressed at an RPK of 111, contained 9 flags in its FDM sequence of which 8 flags were successfully bridged. While correcting 89% of a gene model's detected deviations may be considered significant progress towards correctly determining a gene model's structure, the result is still considered a failure for the purpose of method validation. The over all frequency of deviations is also under-represented in this analysis as only internal flags in the FDM sequence were considered.

This analysis illustrates that at all expression levels considered, many observed deviations between expressed sequence and predicted model can be corrected by this method. Some of those deviations in expression from the model can be attributed to low or uneven depth of coverage, while other deviations might be attributable to errors in the gene model. Deviations due to low sequence coverage will not be corrected by this method. At higher expression levels, the number of zero-flag FDM sequences after correction approaches 100% of the expressed models, presumably in part because at higher expression deviations due to poor sequence coverage occur more infrequently and the majority of observed deviations are due to correctable errors in the predicted gene models. The number of expressed genes however also drops considerably with increasing expression thresholds.

For method validation, a conservative threshold of 500 RPK (an average depth of coverage of about 23) was selected. This threshold is considerably higher than the standard required for genomic sequencing but provides reasonable sequence coverage for gene with nonuniform transcript coverage, In addition, the alignment parameters for Bowtie resulted high confidence alignments and minimize alignments containing insertions or deletion. As will be illustrated, the stringent alignment parameters and high redundancy are essential for identification of sequence deviations and sequence reconstruction. At this expression level, over 95% of bridged FDMs were without internal flags and about 10% of total predicted best gene models are expressed in our RNAseq data set. Even at an average depth of coverage of 3 however, 70% of expressed gene models could be successfully validated or corrected by our proposed method, indicating utility even at relatively low levels of gene expression. If the principle restriction on applicability of this method is depth of coverage, then additionally collected expression data is all that is required to validate and correct larger proportions of the expressed gene models.

### Structural Annotation of Differential Expressed Mycorrhizal Genes

To validate the methods proposed here, we selected a dataset that fit three criteria: 1) the set must be sufficiently large to provide reasonable assurance of method validation; 2) the selected set must be small enough that a researcher could usefully consider each individual result; and 3) the selected set must be composed to be of general, biological interest. We validated our tools for evaluation and improvement of structural annotation using set of gene models significantly differentially expressed mycorrhizal relative to the free-living mycelial fungus, previously described [12]. We identified 1501 gene models from the differentially expressed set with a transcriptomic sequence coverage of 23 or higher. To verify the biological relevance of this mycorrhizal subset (Myc-BestModel), we evaluated the GO-assigned functional annotation of the statistically enriched expressed gene set (Table 1). The Myc-BestModel gene set is enriched for the GO-functional annotations of carbohydrate metabolism (GO:0005975) indicative of the shift in global metabolism. This transition is consistent a shift from environment-derived nutrients to a symbiotic state in which the fungus utilizes simple sugars provided by the plant symbiont. The enriched annotations for membrane (GO:0016020), electron transport (GO:0006118), H(+)-transporting two-sector ATPase, and ATP synthesis coupled proton transport (GO:0015986) are consistent with the changes occurring in transport across the membrane between a free living state drawing all of it required nutrients from its environment to a symbiotic state. The highly enriched annotations associated with protein biosynthesis (GO:0006412, GO:0003735, 0006511, and GO:0005839) are consistent with the significant shift in protein expression between the free living mycelial and mycorrhizal states. These sets of enriched annotations suggest that the 1501 gene models in the Myc-BestModel subset are of potential biological interest and are specific to the changes in *L. bicolor* that accompany the shift from a free living mycelium to symbiotic mycorrhizal transcriptome.

**Table 1:** Enriched Annotations in Myc-BestModels.

Branch	Annotation	pVal*
GO-bp	protein biosynthesis_GO:0006412	0
GO-mf	structural constituent of ribosome_GO:0003735	0
GO-cc	membrane_GO:0016020	2.68E-06
GO-bp	electron transport_GO:0006118	2.04E-06
GO-bp	carbohydrate metabolism_GO:0005975	5.21E-06
EC-def	Proteasome endopeptidase complex.	3.19E-13
GO-cc	proteasome core complex (sensu Eukaryota)_GO:0005839	7.27E-12
GO-bp	ubiquitin-dependent protein catabolism_GO:0006511	2.15E-07
EC-def	H(+)-transporting two-sector ATPase.	8.76E-11
GO-bp	ATP synthesis coupled proton transport_GO:0015986	0.000528

* Gene models annotations in the set of Myc-BestModel that are significantly enriched (p<0.001), with at least 10 gene models with a specific annotation.

### Analysis of Differentially Expressed Mycorrhizal Genes

**Figure 3:**
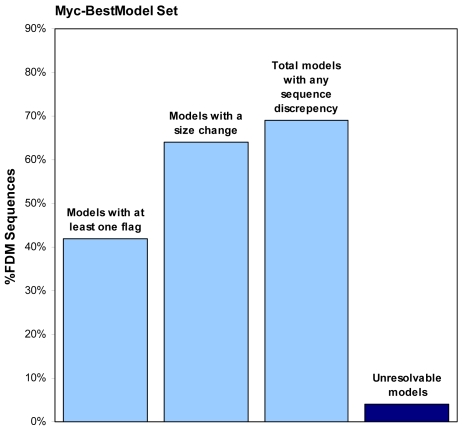
Summary of structural annotation outcome for the Myc-BestModel set.

The transcriptomic data provide an independent approach for both validation and correction of the gene models. The initial phase of the process identifies regions where the transcriptomic sequence deviates from the gene model and also validates intron exon boundaries incorporated in the gene models. For the Myc-BestModel subset of 1501 genes, application of the analysis method resulted in the validation of ∼80% of all intron-exon boundaries (>6000) within the limits of the gene models. The transcriptomic sequence depth of 23 provides a high level of confidence for the quality of these models. This level of validation for the original gene models is notable in view of the complexity of the fungal genome (*L. bicolor* genes contain an average of 5.4 introns) and the annotation limitations arising from the relatively small number of sequenced fungal genomes, especially basidiomycetes. However, the combination of the error rate and intron density means that 42% of the gene models in the mycorrhizal set contain sequence deviations that largely represent intron/exon boundaries that do not map to the mRNA sequence data (Figure 3). For 465 (31%) of the Myc-BestModels in the original best gene model set, we did not detect any inconsistencies and therefore have independently confirmed the previously published BestModelv1 annotation.

Additional analysis scripts enable delineation of the 5′ and 3′ boundaries sequences as defined by the transcriptomic data. The impact of changes at the gene model sequence boundaries is qualitatively assessed by analysis of the change in the size of the gene models. For this analysis, we considered only those models where the sequence length of transcript-derived model differed by greater than 10% (plus or minus) from the initial Myc-BestModel. These criteria result in an observed absolute change in expressed gene size for ∼62% of the Myc-BestModel gene model set. This observation demonstrates that delineation of the boundary sequences results in modification of a significant number of the Myc-BestModel sequences.

To improve the experimental utility of the gene model set, we developed algorithms that use the RNA-seq data to extend the boundaries of the current gene model set where appropriate, identify those intron-exon boundaries that can be validated by the transcriptomic data, and generate novel intron-exon boundaries to bridge those regions of the gene models that are not supported by RNA-seq data. The BES sequences represent the complete, expressed gene sequence but do not themselves have the structural annotation of the gene. This extended and bridged contiguous expressed sequence was then aligned to the genome using a modified Smith-Waterman algorithm to recover gene model's structural annotation. Of the set of 1501 gene models, 1439 (96%) generated modified gene models from BES's in which all error flags were successfully spanned and sequences aligned to genomic sequence. The remaining 62 (4%) of gene models either had error flags that could not be spanned or generated BES's that did not align to genomic sequence. If we consider a gene model significantly changed if it had at least one flag in the FDM sequence that was successfully bridged and aligned to scaffold, a change in the total number of exons, and/or an absolute change in expressed gene size of more than 10%, then of the set of 1439 aligned BESs, 974 (69%) of gene models required changes to match gene expression. 465 (31%) of BESs were not significantly changed, confirming the previously published BestModelv1 annotation.

For the 974 significantly modified gene models, it is useful to look more closely at the nature of the modifications made. Considering the FDMs and counting the number of deviations in expression from the model, 228 (23%) of the 974 significantly modified models had at least one error flag not at the ends of the sequence and 62 (6%) had more than one error flag. 247 (25%) of gene models had error flags at one end of the sequence or both. 582 (40%) of gene models had at least one error flag, either at a terminus or in the middle of sequence. Overall, the average change in gene model size was 27.9%, a substantial general increase in the sequence length of the Myc-GeneModel set, although not necessarily a change in the translated sequence. 594 (61%) of gene models showed a more than 10% increase in gene size while only 28 (3%) of validated gene models showed a more than −10% change in gene size.

Overall, there was an average increase of 0.5 exons added per gene model. Within the initial boundaries of the BestModelv1 genes, however, there was an average −0.1 changes to the number of exons, indicating that most of the increase in number of exons derived from the up and downstream sequence added to the gene model. 339 (35%) of modified gene models showed a net increase in total exons while 126 (13%) showed a net decrease. It is important to note that comparing the number of exons in the BestModelv1 set and in the aligned BESs may be a significant underestimation of the changes made to the BestModelv1. It is possible, for example, that an exon is removed from the model and a new exon added, making the net change in number of exons zero, in spite of the fact that significant changes to the gene model's structure were made. A case in point is the gene model encoding Protein ID 294735. Transcriptomic sequence supports a single intron while the original gene model contained three introns. Similarly, the gene model encoding Protein ID 294625 contained seven introns while the transcriptomic sequence supports a model with 12 introns.

## Discussion

Deep RNA sequencing of the *L. bicolor* transcriptome provides a resource for validation and improvement of structural gene models. This approach is an extension of the classical approach to utilize EST libraries to seed and define gene models [8], . The two approaches are complementary and there is a general consensus for structural annotation derived using the EST or RNA-seq data [21]. However, transcriptomics using next generation sequence technology provide superior coverage and redundancy of the transcriptome [22], [23]. Although the available EST libraries for *L. bicolor* contain approximately 20K clones, the amount of sequence data from the single transcriptomic data set generates 30-times more sequence data than the combined EST reads. The combination of reads derived from a single set of transcriptomic data (30 million reads) enables comprehensive examination of ∼3000 of the gene models in the Bestmodelv1 set using a transcript sequence coverage depth of 23. The sequence coverage depth of 23 was empirically selected to enhance gene coverage and thereby maximize the number of gene with corrected deviation. At this coverage depth, a large fraction of the models are improved by application of algorithms and using the transcriptomic sequence. Even minor changes in the intron/exon boundaries can be resolved by analysis of aligned sequences. A large fraction of sequence discrepancies can be resolved by adjustment of the requirement for transcript sequence coverage depth and inclusion of additional gene models from the BestModelv1 set. Decreasing the sequence coverage depth will expand the number of Bestmodelv1 gene model sequences included in the analysis set and lead to the identification of additional deviations from the model sequence. However, nonuniform transcript distribution will results in coverage gaps for comparison of the gene models with the transcriptomic sequence. A qualitative assessment of relative variability in transcript coverage can be obtained by inspection of transcript coverage for gene models from the corrected set (e.g. Gene model 143003, accessible via the URL provided in the data resources file provided in Supporting Data).

Comparison of the transcript and Myc-BestModel subset sequences led to changes in 974 (69%) of gene models. The large fraction of gene models with revised structural annotation is not perhaps surprising in view of the relative complexity of the target set. The set of best models used for analysis was generated primarily by an automated annotation process [12]. This process effectively identified gene candidates and requires little manual input. However, this homology-based process often results in unresolved structural features and a limited ability to associate relevant biological information with the sequences [1], [6]. Current approaches for structural annotation are unable to accurately define exon content or location [24], [25] for many genes. Analysis of internal sequence deviations in gene model sets from different annotation methods indicates a variance in the frequency of internal sequence deviation in gene model sets derived by different annotation methods (Supplemental Data Table S1). In general, EST-based prediction methods tend to have a lower percentage of models with internal FDMs. However, there is a wide variance in the number of models derived by the methods. The presence of a significant number of short exon segments in basidiomycetes further increases the complexity of the structural annotation process for *L. bicolor*. In spite of these impediments, the sequence depth and ease of data generation indicate RNA sequencing provides a reliable resource for validation and improvement of structural gene models.

### Corrections to the Gene Models

The transcriptomic sequence provides the opportunity for accurate identification and localization of exons and enables the verification or correction of the structural annotation. Our targeted approached leverages previously collected information to find splice sites that match observed data and focuses computational effort only on those splice sites that are not supported by RNAseq data. Analysis of internal gene model deviations suggest 42% of the expressed and selected set of *L. bicolor* gene models contain sequence deviations that largely represent intron/exon boundaries that do not map to the mRNA sequence data (Figure 3). Our method has no restrictions on intron lengths and can identify exons as short at 9 bp. The method also places no expectations on splice donor and acceptor sites. Unlike other published methods, our method has an advantage for identification of splice sites even if they are not a previously reported or observed. This strategy reasonable at high sequence coverage depth and enable identification of the maximum number of exons. A drawback of this approach is increased ambiguity of the exact genome location of the splice site. Our alignments of the transcript-derived models to the genome can lead to the improper placement of the final coding nucleotide associated with the beginning or end of an exon. This can be resolved by manual inspection of the gene of interest or the output of this method can also be used in conjunction with other splice assembly programs [8].

**Figure 4:**
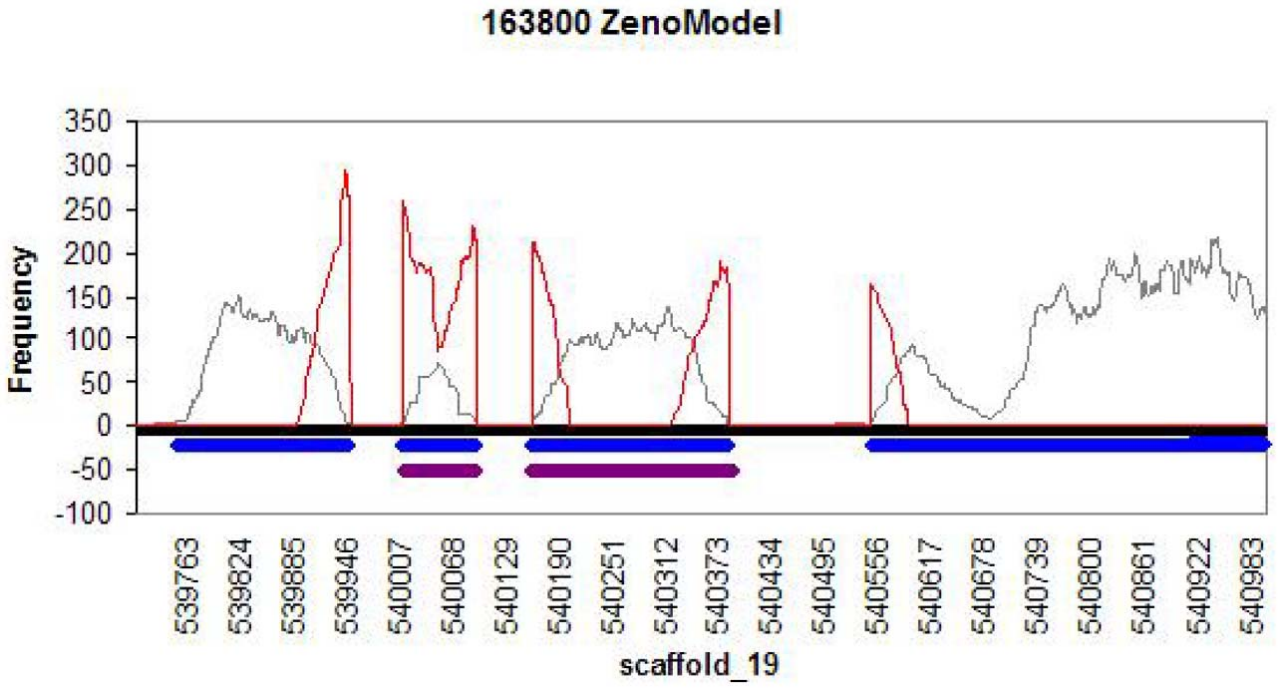
Comparison of the original and revised gene models encoding protein 163800 on genome sequence scaffold 19. Location on scaffold is on x-axis and numbers of read alignments at each bp is on the y axis. The gray line represents alignments to genomic sequence, The red lines are read alignments to JGI gene model and corrected gene model, but not to the genomic sequence. The sequence span of original gene model is displayed a green horizontal bar at the bottom of the transcript coverage trace. The revised gene model is displayed as a blue horizontal bar.

One additional interesting category of modified gene models are those which contain no deviations from observed expression data within the boundaries of the BestModelv1 gene models, but nonetheless showed significant changes to gene structure by addition of up and/or downstream expressed sequence. A case in point is the gene model encoding protein ID 163800. The initial model consisted of two exons and did not contain an appropriate start methionine (Figure 4). The revised model is more than doubled in size, contains four exons, and candidate start codons. Of the 974 modified gene models, 453 (30%) had no error flags within their FDM sequences, but showed a more that 10% increase in gene size. Of these 453 expanded genes, 193 (43%) also showed in increase in the total number of exons. Changes that enabled definition or extension of the N- or C-termini are especially informative to enable expression and provide clues for function. Modification of the N- or C-terminus can result in the identification of additional sequence features such as signal peptides, regions with membrane spanning helices or pfam domains. This information is useful for functional annotation and is often essential to enable *in vitro* protein expression and future biochemical characterization. Substantial changes to predicted UTRs also affect the ability to predict the regulatory mechanisms of mycorrhizae-specific genes.

### Uncorrected models

A vast majority of the Myc-BestModel gene models (1439 or 96%) generated modified gene models with all error flags were successfully spanned and transcript sequences well aligned to genomic sequence. The remaining 62 (4%) of gene models either had error flags that could not be spanned or generated BES's that did not align to genomic sequence. The quality of the genome scaffold contributes in part to the inability to reconcile these gene models is attributable to genome correct some of this sequence lies in the incomplete nature of the genomic sequence. The genome is currently contained in 1178 scaffolds with some unresolved nucleotide regions. Several gene models contain regions of unresolved sequence (e.g. N nucleotide designations) in middle of gene sequence (e.g. gene models coding for the 293291 or 298293 proteins) and/or unusually long introns regions (e.g. the model coding for the 298293 protein). For several genes, the presence of alternative transcripts resulted in an uncorrected model (e.g. the model coding for the 247279 protein) indicating the method may not identify multiple splice variants when more than one variant is simultaneously present in dataset. For this study, we restricted the RNA-seq data set to a single biological condition to minimize the contribution of alternative transcripts and facilitate the methods development and evaluation process. The current method will generate a corrected gene model if one of the variant represents >50% of all transcript sequences at the site of the alternative splice. The gene model encoding the 308888 protein contains five exons with clearly identified bridging sequences spanning each of the introns (accessible via the URL provided in the data resources file provided in Supporting Data). However, inspection of the transcript map reveals the presence of transcripts that likely span this intron and likely represent a read through intron variant. The identification of splice variants associated with the free living, interaction, and mycorrhizal states may have implications for the regulation of metabolic, functional, and metabolic capabilities. In view of the biological relevance of these genes, we are developing methods for identification of splice variants using RNA-seq data. For several uncorrected models, reasons for failure were not immediately obvious and will likely require extensive biological confirmations to resolve which is beyond the scope of this study.

High-throughput transcriptomic data was found to provide far greater resolution for resolving gene structure than a previously collected data set of ESTs. The outcome of this process is a set of high confidence gene models that can be reliably used for experimental characterization of protein function. The corrected gene structures improve capabilities for the prediction of protein function and are required for *in vitro* approaches to characterize the function of these proteins. The method described here can be applied to improve gene structural annotation in other species, provided that there is a sequenced genome and a set of gene models. This improved annotation process can be extended to other important gene families and will facilitate the process to identify the molecular mechanisms leading to the development of the mycorrhizal symbiosis and its implications in improving carbon sequestration by poplar.

## Methods

### Laccaria bicolor culture, *In vitro* interaction setup and RNA extraction

*L. bicolor* (Maire) Orton (strain S238N) culture was maintained on Modified Melin Norkan's media as described [26] at 20°C. Total RNA was extracted from control, interaction and mycorrhizal samples by CTAB method [27]. Total RNA is treated with RQ-DNase (Promega, Madison, WI).

### mRNA-Seq sample preparation

Procedures described for preparation of mRNA for the mouse transcriptome analysis [28] were used with some modifications. Ten ug of total RNA from each was hybridized to Sera-mag oligo (dT) beads (Thermo Scientific) for mRNA purification. Purified mRNA was fragmented by addition of 5× fragmentation buffer (Illumina, Hayward, CA) and was heated for 5 min at 94°C in a thermocycler. First strand cDNA was synthesized using random primers to eliminate the general bias towards 3′ end of the transcript. Second strand cDNA synthesis was done by adding GEX second strand buffer (Illumina, Hayward, CA), dNTPs, RNaseH and DNA polymerase I followed by incubation for 2.5 h at 16°C. Second strand cDNA was further subjected to end repair, A-tailing, and adapter ligation in accordance with the manufacturer supplied protocols. Purified cDNA templates were enriched by PCR amplification with Phusion DNA polymerase (Illumina, Hayward, CA) and the samples were cleaned using QIAquick PCR purification columns and eluted in 30 µl EB buffer as per manufacturer's instructions (QIAGEN, CA). Purified cDNA libraries were quantified using Nanodrop spectrophotometer and loaded onto Illumina flow cells. A total of three sequencing lanes were run. One lane was run to 46 bp reads, the others were run to 72 bp reads. For this study, all 72 bp reads were truncated to a uniform 46 bp by removal of bases from the 3′ end. The total sequence yield was 39.1 million reads.

### Gene Models and *L. bicolor* Genomic Sequence

*L. bicolor* genomic sequence and gene models were taken from Joint Genome Institute, using publically available data files. Best Modelsv1 is the filtered set of models representing the best gene model for each locus from the Joint Genome Institute (JGI). “BestModels1.na.fasta”, contains 20614 gene models which average 1.1 Kbp per gene with an average of 5.4 exons of an average 210 bp per exon [12]. The intron density and average intron length is consistent with that observed in other basidiomycetes. In the BestModelsv1 set, 92% of the splice donor sites are GT and 5% GA. AG and GC comprise 94% and 5% of the splice acceptor sites, respectively.

### Identification of *L. bicolor* Mycorrhizal Transcriptome

Affymetix data from [12] was used to identify genes differentially expressed in mycorrhizal L. bicolor relative to free-living mycelial *L. bicolor*. Samples compared were “Laccaria/Populus ECM greenhouse” and “Laccaria/Populus ECM in vitro” vs. “Free-living mycelium Laccaria bicolor S238N 1” and “Free-living mycelium Laccaria bicolor S238N 2”

We selected 1501 gene models expressed at greater than 500 RPK from Set 3 of the ‘Bowtie’ alignments. Gene models in the selected set were differentially expressed in Mycorrhizal vs. Free Living Mycelium at a absolute value log 2 fold change greater than 0.6 (a minimum 1.5 fold change) at a Benjamini-Hochberg (BH) [29] False Discovery Rate (FDR) corrected Local Pooled Error (LPE) test [30] p-value <0.05.

### Biological significance of *L. bicolor* Mycorrhizal Transcriptome

To assign a p-value to observations that can be considered as the probability that some number of successes is observed in a set of observations, the Cumulative Binomial Distribution (CBD) can be used. The binomial probability mass function is written as:
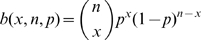
The cumulative binomial distribution is a function of the binomial probability mass function:
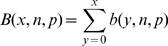
The CBD as written here returns the probability that there are at most number *x* successes in *n* trials where each trial has a probability of success *p*. A CBD-derived p-value is reported as 1-*B*. For determining the significance of enrichment of annotations in the set of 1501 gene models (myc-BestModels) representative of the mycorrhizal transcriptome, The enrichment of a specific annotation is determined as CBD p-value with *x* equal to the number of gene models in the transcriptome set with a that specific annotation, *n* equal to 1501, and *p*, the probability of success equal to the fraction of BestModelsv1 with that annotation.

### Alignment of RNA-seq reads to gene models and genomic sequence

The ultra-fast sequence alignment program “Bowtie-0.9.9” [19] was used to generate alignments of RNA sequence reads to the genome and gene models. “Bowtie” uses a Burrows-Wheeler index to rapidly align sequences to a pre-processed indexed set of sequences and uses a small memory footprint. The output of “Bowtie” includes the names of the reads that aligned, the orientation of the reads in the alignments, the names of the sequence to which the reads aligned, and the offsets into the reference sequences of the alignments. The default Bowtie conditions of a default high-quality read length of 28, two permitted mismatches in the high-quality end of a read, and maximum acceptable quality distance of the overall alignment = 70 were used to generate the following subsets of sequence alignments from the total 39.1 million collected RNA-seq reads:

**Set 1** – RNA-seq reads aligned to genomic sequence “laccaria.allmasked” (15.4 million reads aligned)
**Set 2** - RNA-seq reads cDNAs aligned to gene models “BestModels1.na.fasta” but not to genomic sequence (3.7 million reads aligned)
**Set 3** - RNA-seq reads cDNAs aligned to gene models “BestModels1.na.fasta” (16.9 million reads aligned)

### Identify deviations in RNA-seq data from gene models

A Perl script was written to analyze the output from ‘Bowtie’ to determine how many aligned reads were found for every base pair on the scaffolds how many reads aligned to the proposed splice sites of each gene. The script parameters were initially validated using a subset of 1501 genes associated with the mycorrhizal transcriptome. The gene models in this subset of the Bestmodels were used to validate the ability to find and correct discrepancies between the predicted gene models and the observed transcriptomic data. The complete method is also summarized in Figure 1.

For each gene model analyzed, a Flagged Deviations from Model (FDM) sequence is generated by the following method.

For every gene, indexed *n*, in analysis set
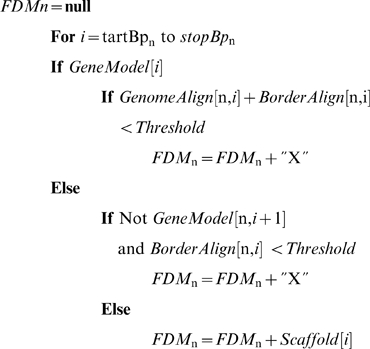

*startBPn* and *stopBPn* are the start and stop positions of the gene model on the scaffold for gene *n*. *GeneModel*[*n*, *i*] is an array, indexed by scaffold bp equal to **TRUE** when the base pair at scaffold position *i* belongs to the gene model n, and equals **FALSE** otherwise. *GenomeAlign*[*i*] is the number of RNA reads from Set 1 ‘Bowtie’ alignments to scaffold at position *i*. *BorderAlign*[*i*] is the number of RNA reads alignments from Set 2 ‘Bowtie’ alignments to scaffold at position *i* that align to the gene model but not to the scaffold, indicating a read that spans a splice site. *Scaffold*[*i*] is the positive strand base (A, G, C, T, or N) at position *i*. *FDMn* is the Flagged Deviation from Model sequence for gene n. *Threshold* is the minimum number of read alignments required to consider there to be expression. For this study, a Threshold of 2 alignments was used. A base pair of “X” here is used as an error flag in the FDM sequence, identifying regions in the gene model not supported by the expression data and that require correction. The resulting *FDMn* is the sequence for the gene model n as it is expressed in the set of RNA-seq data with error flags inserted into the sequence where the observed expression was found to deviate from the published gene model.

### Bridge potential model errors with assembled contigs of RNA-seq reads using FDMs as starting points

For each FDM generated above, identify regions of gene model not supported by RNA-seq data. Concatenate any sets of multiple, contiguous error flags into a single flag and remove from FDM any sets of sequences less than 36 bp long between error flags and concatenate into a single Error Flag. For every error flag in FDM, identify an expressed 18-mer probe sequence, up and downstream of the error flag. The contig is identified, assembled between the upstream and downstream probes using the method described below, and the transcript derived sequence is used to replace the error flag.

Error flags at the beginning and end of the FDM are noted, but removed from the sequence. Being at the ends of the sequence, probes up and downstream from the expressed portions of the gene model cannot be generated. Using a slight modification of the method used to bridge error flags, the FDM is extended upstream and downstream of its initial boundaries if possible. The computational method for bridging and extending gene models, “Bridge” is described in detail below. The result of the FDM with bridged error flags and extensions up and down stream of the initial gene model boundaries is new sequence saved as a Bridged and Extended Sequence (BES).

### Identify Bridging Contigs between probe sequences

RNA-seq data was used to extend and bridge errors in gene models with the following procedure, “Bridge”. Bridge accepts two probe sequences as input and searches the set of RNA-seq data for a continuously expressed spanning sequence, if any, that connects the two probe sequences.
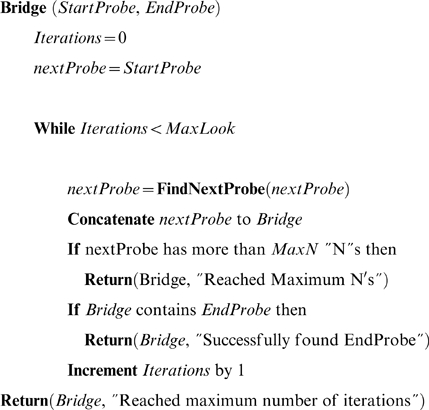

*StartProbe* and *EndProbe* are the probes upstream and downstream of a flagged deviation from model respectively. *Bridge* is the variable, initialized to an empty string, that stores the identified expressed contiguous sequence between *StartProbe* and *EndProbe*. *MaxLook* is the maximum number of iterations allowed and *MaxN* is the maximum number of unknown ‘N’ base calls in the assembled bridge permitted before the procedure terminates. The function **FindNextProbe** is described below. This function returns the identified and expressed consensus sequence that bridges the *StartProbe* and *EndProbe* sequences. As it is possible the procedure may not identify a bridging sequence, the procedure also returns a message describing why the search for the bridge sequence was terminated. In this study, to bridge probes that spanned errors in FDMs, *maxLook* was set equal to 27 and *maxN* equal to 6. To extend the gene sequences past its initial borders, Bridge is called without a value for *EndProbe*, and *maxN* equal to 1.

The procedure “FindNextProbe” used in “Bridge” is used to search the set of collected RNAseq reads for consensus sequence and finds the next probe in a growing contig sequence.
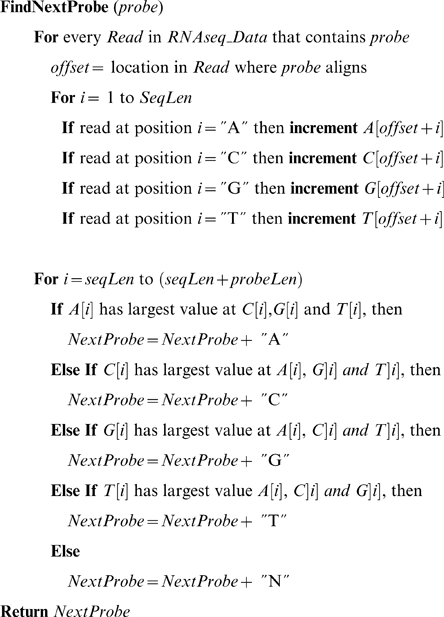
Where *probe* is the sequence of length *probeLen*, and *RNAseq_Data* is the set of all sequence reads. *SeqLen* is the length in base pairs of the RNA-seq reads used in the analysis. Arrays *A*, *G*, *C*, and *T* store the number of instances of each base at each position in the consensus sequence being constructed and all arrays are initially populated with zeros. The function returns the next *probeLen*-sized sequence from the assembled consensus sequence. For spanning a flagged region of the FDM, the first probes are selected starting 6 bp up and downstream of the flag. If these probes do not identify a spanning sequence, then new probes 15 bp up and downstream of the flag are used. If these probes fail, probes 21 bp up and downstream of the flag are used. If these return no spanning sequence, then the error is flagged as unidentified. Although a perfect match is required for finding up and downstream probes, this step-wise procedure will identify spanning sequence even if there are mismatches in one of the possible probes. Also, this procedure can accommodate for possible misalignments at the intron-exon border where some alignments occur that crosses by a few base pairs the intron-exon boundary. When extending the FDM up and downstream, the first and last 18 bp of the FDM are used as the initial probes.

### Align BESs to scaffolds

The BESs, constructed from RNA-seq data corrected FDM's represent the expressed gene sequence as transcribed under specific biological conditions, but they do not contain information regarding the gene structure. Assembled sequence that bridges an identified error in a BES might contain only corrected intron boundaries, or might introduce a number of previously unreported exons. In order to recover gene structure information, it is necessary to align the BES back to the *L. bicolor* chromosome. This was accomplished using the Smith-Waterman algorithm with a few minor modifications [31]. The *L. bicolor* scaffold sequences were taken from “Laccaria.alllasked” from JGI.

The penalty for inserting a gap into the chromosomal sequence is −24 while the gap penalty for inserting a gap into the BES gene sequence is −1. The penalty for inserting a gap at the beginning or end of the BES is 0. This fits our expectations for the alignment. Gaps in the chromosome are unlikely, but not impossible, suggesting that some erroneous deletion from the assembled scaffold has been made. Gaps in the BES sequence, representing introns, are expected and so the penalty is less than for the scaffold sequence. Match-Mismatch scores were set to 8 for a match, −12 for a mismatch, and 4 for any nucleotide matching with an ‘N’. ‘N’ nucleotides can be present both in the published genomic sequence and as a consequence of the bridging contig method described below. A score of 4 for an ‘N’ prevents inserting spurious gaps in the contig sequence without over-fitting contiguous ‘N’s into the alignment. Though *L. bicolor* exons can be short, isolated alignments of small sets of nucleotides bracketed by spaces in the BES should be discouraged. In our modified Smith-Waterman algorithm, alignments of less than 4 contiguous nucleotides in the BES, allowing an ‘N’ to match any nucleotide, are not permitted.

Before alignment, 5% off the end of any sequence added from the gene model extension, upstream or downstream, was trimmed. The sequence of the scaffold selected for alignment is the region within the original BestModelv1 boundaries plus three times the size of the up and downstream sequence added. If the first attempt to align the BES to the genomic sequence failed, the sequences were reversed and complimented and a second attempt to realign was made. From the alignment, the gene model's structural annotation can be generated and was saved in GFF format. The final output of this method is the set of expressed BES gene models in FASTA format, the structural annotation of the gene models in GFF format, and a new set of FDMs sequences resulting from the re-alignment RNA-seq data to the modified gene models using ‘Bowtie’.

## Supporting Information

Text S1 A gff format text file containing all revised gene models for the Myc-BestModel gene model set.
(0.74 MB TXT)

Text S2 A fasta format text file containing the modified transcript sequences for the Myc-BestModel gene model set.
(1.85 MB TXT)

Text S3 A text file listing project data files and resources available for viewing and download. A project URL is provided to facilitate access to these resources.
(0.00 MB TXT)

Table S1 Comparison of Internal Sequence Deviations in Gene Model Sets from Different Annotation Methods.
(0.09 MB DOC)
