# Supplementary material for: Using Deep RNA Sequencing for the Structural Annotation of the Laccaria Bicolor Mycorrhizal Transcriptome
Source: PLoS One. 2010 Jul 6;5(7):e9780. doi: 10.1371/journal.pone.0009780 (PMC2897884; doi:10.1371/journal.pone.0009780)
Supplement: Table S1 — Comparison of Internal Sequence Deviations in Gene Model Sets from Different Annotation Methods. (0.09 MB DOC) [file pone.0009780.s004.doc]

| **ANNOTATION METHOD** | **# Gene Model** | **# Models with FDM** | **% Models with FDM** |
| --- | --- | --- | --- |
| ***All Expressed Gene Models*** |  |  |  |
| e_gwh1 | 314 | 175 | 56% |
| e_gww1 | 528 | 338 | 64% |
| estExt_fgenesh1 | 56 | 2 | 4% |
| estExt_fgenesh2 | 1079 | 524 | 49% |
| estExt_Genewise1_human | 95 | 20 | 21% |
| estExt_Genewise1_worm | 70 | 22 | 31% |
| estExt_GeneWisePlus_human | 278 | 89 | 32% |
| estExt_GeneWisePlus_worm | 394 | 172 | 44% |
| eu2 | 3050 | 1287 | 42% |
| fgenesh3 | 603 | 412 | 68% |
| gwh1 | 214 | 94 | 44% |
| gww1 | 322 | 199 | 62% |
|  | 7003^*^ | 3334 | 48% |
| ***Myc-BestModel gene set*** |  |  |  |
| e_gwh1 | 72 | 22 | 31% |
| e_gww1 | 103 | 53 | 51% |
| estExt_fgenesh1 | 35 | 0 | 0% |
| estExt_fgenesh2 | 305 | 69 | 23% |
| estExt_Genewise1_human | 0 | 0 | 0% |
| estExt_Genewise1_worm.C | 76 | 10 | 13% |
| estExt_GeneWisePlus_human | 105 | 13 | 12% |
| estExt_GeneWisePlus_worm | 141 | 35 | 25% |
| eu2 | 493 | 67 | 14% |
| fgenesh3 | 77 | 29 | 38% |
| gwh1 | 40 | 9 | 23% |
| gww1 | 53 | 15 | 28% |
| All JGI Models | 1500^*^ | 322 | 21% |

^*^Not all gene models are represented in the listed categories
